# The Bittersweet Symphony of COVID-19: Associations between *TAS1Rs* and *TAS2R38* Genetic Variations and COVID-19 Symptoms

**DOI:** 10.3390/life14020219

**Published:** 2024-02-03

**Authors:** Aurora Santin, Beatrice Spedicati, Alessandro Pecori, Giuseppe Giovanni Nardone, Maria Pina Concas, Gioia Piatti, Anna Menini, Giancarlo Tirelli, Paolo Boscolo-Rizzo, Giorgia Girotto

**Affiliations:** 1 Department of Medicine, Surgery and Health Sciences, University of Trieste, 34149 Trieste, Italy; aurora.santin@burlo.trieste.it (A.S.); beatrice.spedicati@burlo.trieste.it (B.S.); giuseppegiovanni.nardone@burlo.trieste.it (G.G.N.); tirellig@units.it (G.T.); paolo.boscolorizzo@asugi.sanita.fvg.it (P.B.-R.); giorgia.girotto@burlo.trieste.it (G.G.); 2 Institute for Maternal and Child Health, I.R.C.C.S. “Burlo Garofolo”, 34137 Trieste, Italy; alessandro.pecori@burlo.trieste.it; 3 Department of Pathophysiology and Transplantation, University of Milan, 20122 Milan, Italy; gioia.piatti@unimi.it; 4 Unit of Bronchopneumology, Fondazione IRCCS Ca’ Granda Ospedale Maggiore Policlinico, 20122 Milan, Italy; 5 Neurobiology Group, SISSA, Scuola Internazionale Superiore di Studi Avanzati, 34136 Trieste, Italy; menini@sissa.it

**Keywords:** COVID-19, COVID-19 symptoms, *TAS1R2*, *TAS1R3*, *TAS2R38*, immune response

## Abstract

The innate immune system is crucial in fighting SARS-CoV-2 infection, which is responsible for coronavirus disease 2019 (COVID-19). Therefore, deepening our understanding of the underlying immune response mechanisms is fundamental for the development of novel therapeutic strategies. The role of extra-oral bitter (TAS2Rs) and sweet (TAS1Rs) taste receptors in immune response regulation has yet to be fully understood. However, a few studies have investigated the association between taste receptor genes and COVID-19 symptom severity, with controversial results. Therefore, this study aims to deepen the relationship between COVID-19 symptom presence/severity and *TAS1R* and *TAS2R38* (*TAS2Rs* member) genetic variations in a cohort of 196 COVID-19 patients. Statistical analyses detected significant associations between rs307355 of the *TAS1R3* gene and the following COVID-19-related symptoms: chest pain and shortness of breath. Specifically, homozygous C/C patients are exposed to an increased risk of manifesting severe forms of chest pain (OR 8.11, 95% CI 2.26–51.99) and shortness of breath (OR 4.83, 95% CI 1.71–17.32) in comparison with T/C carriers. Finally, no significant associations between the *TAS2R38* haplotype and the presence/severity of COVID-19 symptoms were detected. This study, taking advantage of a clinically and genetically characterised cohort of COVID-19 patients, revealed *TAS1R3* gene involvement in determining COVID-19 symptom severity independently of *TAS2R38* activity, thus providing novel insights into the role of TAS1Rs in regulating the immune response to viral infections.

## 1. Introduction

Severe acute respiratory syndrome coronavirus 2 (SARS-CoV-2) infection is responsible for the greatest worldwide public health threat of the 21 century, coronavirus disease 2019 (COVID-19) [1,2]. COVID-19 is characterised by a broad spectrum of symptom manifestations and severity, ranging from asymptomatic or mild–moderate forms, whose most common symptoms are nasal obstruction, sore throat, and rhinorrhoea, to severe conditions characterised by symptoms such as fever, cough, fatigue, vomiting, diarrhoea, and dyspnoea [3]. Further, it has also been reported that COVID-19 patients frequently display smell and/or taste dysfunctions, such as a decrease in or loss of olfactory perception and taste sensitivity [4].

As widely demonstrated, the innate immune system has a relevant role in fighting SARS-CoV-2 infection; therefore, a fuller knowledge of the physiopathological mechanisms underlying innate immunity has proven fundamental for the development of novel therapeutic strategies for COVID-19 [5].

A growing body of literature is highlighting the emergent role of bitter and sweet taste receptors in regulating upper respiratory tract immune response [6]. Bitter and sweet taste receptors are a family of G-protein-coupled receptors (GPCRs) first identified in type II taste receptor cells of the tongue [6,7]. Bitter taste receptors (TAS2Rs) detect bitter compounds, acting as a first-line defence mechanism for the human body against the consumption of potentially poisonous compounds that could be present in ingested food, drugs, or bacterial sources [8]. Conversely, sweet taste receptors (TAS1Rs) respond to sugars through the activity of the heterodimeric GPCRs complex encoded by *TAS1R2* and *TAS1R3* genes [9]. Beyond the tongue, *TAS2Rs* are abundantly expressed in the upper respiratory tract [7,10] in two main cellular populations: (1) ciliated cells in which, upon stimulation, they regulate ciliary beating and antimicrobial peptides (AMPs) secretion [11]; (2) in co-expression with *TAS1R2/3* genes in solitary chemosensory cells (SCCs) [11], which are specialised nonciliated chemoresponsive cells involved in innate immune response regulation [12]. 

Activation of TAS2Rs on SCCs by bitter compounds, such as AHLs, triggers AMPs secretion and the activation of downstream signalling components (e.g., α-gustducin, PLCβ2, TRPM5, and IP3R3), thus triggering the inflammatory cascade [13].

Interestingly, recent studies have demonstrated that TAS2Rs and TAS1Rs present on SCCs act in an antagonistic, glucose-dependent manner to regulate the upper airway’s innate immune responses [14]. In particular, in healthy conditions, TAS1Rs activation mediates the suppression of TAS2Rs activation, thus preventing continuous AMPs secretion that may be harmful for the upper airway’s epithelium. 

Conversely, during infections, TAS1Rs, after being activated by bacterial consumption of airway surface liquid glucose, diminish TA2Rs suppression, thus allowing AMPs secretion and triggering a proper immune response [13]. In fact, as demonstrated, sweet substances in the upper airway’s surface liquid inhibit TAS2Rs-induced calcium release, thus impairing calcium-mediated triggering of the innate immune response [13]. 

One of the most peculiar and frequently studied members of the TAS2Rs family is the bitter taste-sensing type 2 receptor (*TAS2R38*) gene. In contrast to other *TAS2Rs* members, *TAS2R38* is expressed only in the ciliated cells of the sinonasal cavity [15], and it is able to specifically respond to acyl-homoserine lactones (AHLs) family’s bitter compounds (e.g., phenylthiocarbamide (PTC), propylthiouracil (PROP) and other chemically similar substances) [16]. Indeed, the binding of AHLs to TAS2R38 triggers the release of AMPs and nitric oxide (NO) [17], which is reported to inhibit SARS-CoV virus replication [18].

In humans, there are two predominant high-frequency *TAS2R38* gene haplotypes, determined by three single-nucleotide polymorphisms (SNPs) (i.e., rs713598, rs1726866, and rs1024693) [19]: (i) the proline–alanine–valine (PAV) haplotype, encoding the functional TAS2R38, and the (ii) alanine–valine–isoleucine (AVI) haplotype, encoding the non-functional variant of *TAS2R38* [13]. These haplotypes dictate the phenotypic variability in phenylthiocarbamide (PTC) and propylthiouracil (PROP) taste sensitivity, enabling classification of the worldwide population as (i) *supertasters* (homozygous PAV/PAV individuals); (ii) *tasters* who perceive different levels of bitter intensity correlating with the relative expression levels of PAV and AVI alleles; (iii) *nontasters* (homozygous AVI/AVI subjects) [20]. Recent studies revealed that the genetic variability within *TAS2R38* gene determines each individual’s susceptibility to upper respiratory infections; in particular, *nontaster* individuals are exposed to an increased risk of respiratory tract infections [21] compared to PAV/PAV individuals [22].

On the other hand, the role of *TAS1R2* and *TAS1R3* in regulating the upper airway’s innate immunity is poorly elucidated. However, among *TAS2Rs*, several SNPs regulate sweet taste receptor activity, thereby influencing each individual’s sensitivity to sweet compounds [23]. In particular, a missense of the SNPs within *TAS1R2,* namely rs35874116, has been associated with sugar intake regulation [24], while rs307355 and rs35744813, located upstream of the *TAS1R3* gene coding sequence, as well as being in high *linkage disequilibrium*, are reported to account for the 16% of sweet taste perception variability in European, East Asian and African populations [23]. 

Therefore, considering the peculiar expression of *TAS1R2/3* genes in SCCs and their role in the regulation of TAS2Rs activity, it was hypothesised that genetic variations within *TAS1Rs* genes could also have an impact on the different individuals’ susceptibility to airway infections [11]. Indeed, considering that TAS1Rs negatively regulate TAS2Rs activity, it was speculated that genetic variants within *TAS1Rs* genes could impact the proper activity of these receptors, thus modulating TAS2Rs-mediated AMPs secretion and the consequent immune response.

Therefore, the emergent role of TAS2Rs and TAS1Rs in respiratory tract immune response regulation led to the hypothesis that they could be implemented as novel prognostic biomarkers for respiratory infections, including COVID-19. To date, still little is known about TAS1Rs’ role in mediating SARS-CoV-2 immune response, while a few studies have investigated the relationship between *TAS2R38* activity and COVID-19 symptom severity, with variable results. On the one hand, some studies have described a positive correlation between *nontaster* status and severe COVID-19-related symptomatology [25]; on the other, the only available study in the literature that genetically investigated the *TAS2R38* haplotype in COVID-19 patients reported no correlation between TA2R38 activity and symptom severity [26].

To fill this knowledge gap, this study aims to clarify the entangled association between COVID-19 symptoms and their relative severity and *TAS1R2*, *TAS1R3*, and *TAS2R38* genetic variations, taking advantage of the clinical and genetic data of a COVID-19 patient cohort. Further, for the first time, the relationship between genetic variations within *TAS1Rs* and COVID-19 symptoms is evaluated, thus offering novel intriguing insights into *TAS1Rs’* role in the immune response during viral infections.

## 2. Materials and Methods

### 2.1. Ethical Statement

Written informed consent was obtained from all participants for their participation in the study and for the collection of biological samples for research purposes. The study was conducted in accordance with the Declaration of Helsinki and approved by the Ethics Committee of Friuli-Venezia Giulia Region (Application No. CEUR-2020-Os-156).

### 2.2. Patients Cohort and Clinical Evaluation

A cohort of 196 adult patients affected by COVID-19 (https://www.covid19treatmentguidelines.nih.gov/overview/clinical-spectrum/, accessed on 18 December 2021) was enrolled at the Section of Otolaryngology, University of Trieste (Trieste, Italy), between September 2021 and November 2022.

COVID-19 diagnosis was confirmed with a positive test for SARS-CoV-2 RNA by polymerase chain reaction (PCR) on nasopharyngeal swabs, according to WHO recommendations ([27], https://www.who.int/emergencies/diseases/novel-coronavirus-2019/technical-guidance, accessed on 18 December 2021).

Standardised self-administered validated questionnaires were employed to collect basic demographic and anamnestic data, including comorbidities (i.e., diabetes, active cancer, cardiovascular disease, chronic respiratory disease, and liver disease).

Patients were asked to evaluate COVID-19 symptoms in the acute phase (AP) of infection, employing a structured questionnaire: the Acute Respiratory Tract Infection Questionnaire (ARTIQ). Specifically, the analysed symptoms were dry cough, coughing up mucus, hearing loss, blocked nose, rhinorrhoea, sneezing, lacrimation, hoarseness, fever, sweating, chills, headache, sore throat, muscle pain, joint pain, chest pain, sinonasal pain, swollen glands, loss of appetite, problems breathing, and shortness of breath. The presence of symptoms was registered as a dichotomous variable (1: “yes”/0: “no”) and symptom severity was ranked on a 0–2-point scale as none (0), mild (1), or severe (2) [28].

### 2.3. Sample Collection and Genetic Analysis

For each enrolled patient, a peripheral whole blood sample was collected for DNA extraction and whole-genome sequencing (WGS) analysis. 

Genomic DNA was extracted using the QIAsymphony^®^ SP instrument with QIAsymphony^®^ Midi Kit (Qiagen, Venlo, The Netherlands), following the manufacturer’s instructions. Quality of DNA was checked with 1% agarose gel electrophoresis and concentration was assessed employing the Nanodrop ND 1000 spectrophotometer (NanoDrop Technologies Inc., Wilmington, DE, USA).

WGS was performed using the Illumina NovaSeq 6000 platform with the Illumina DNA Prep Kit (Illumina Inc., San Diego, CA, USA)*,* according to the manufacturer’s protocol. 

After sequencing, FASTQ files were generated and analysed through a standard pipeline. This pipeline comprises several steps, including (1) quality control performed with *Fastqc* software (version 0.11.9) (https://www.bioinformatics.babraham.ac.uk/projects/fastqc, accessed on 20 January 2022), (2) sequence alignment to the Human Genome Reference build 38p.13 (GRCh38p.13) using the *BWA* software (version 2.1) [29], (3) PCR duplicate removal from BAM files using *Sambamba* (version 1.0) [30], and (4) base quality score recalibration using GATK (version 4.1.9.0) [31]. Finally, indexed CRAM files were produced using *Samtools* (version 1.14) [32]. Joint variant calling was performed using GATK *haplotype caller* and *GenomicsDBImport* [31] generating gVCF files.

Afterwards, a quality check was performed applying *Variant Quality Score Recalibration* with *GATK* and the following filter thresholds: Hardy–Weinberg equilibrium (*p*-value < 10^−8^), missing rate (missing rate > 0.05), heterozygosity rate (*p*-value < 10^−8^), coverage (coverage pre variant calling ≥ 20), and singletons distribution (singletons distribution mean >3 standard deviations). 

Moreover, a phasing step was performed using *Eagle* software (version 2.4.1) [33] and data annotation was performed using Ensembl *Variant Effect Predictor (VEP)* version 106 [34]. 

To reach the main goal of the study, employing *Bcftools* (version 1.14) plug-in *split-vep* (https://samtools.github.io/bcftools/howtos/plugin.split-vep.html, accessed on 20 January 2022), the following *TAS1Rs*-related SNPs were extracted from VCF files: rs35874116 for *TAS1R2* [24], and rs307355 for *TAS1R3* [11]. For *TAS2R38* haplotype analysis, the following SNPs were extracted: rs1726866, rs713598, and rs10246939 [19]. Haplotypes were determined using Bayesian imputation (software *PHASE* v2.1.1) [35,36].

### 2.4. Statistical Analysis

Logistic regression models, adjusted for sex and age, were generated to evaluate the association between presence of ARTIQ symptoms and *TAS1R2*, *TAS1R3* SNPs, and *TAS2R38* haplotype. *TAS1R2* and *TAS1R3* SNPs were coded 0 for homozygous reference, 1 for heterozygous, and 2 for homozygous alternative, while *TAS2R38* haplotype was considered a 3-level factor (AVI/AVI, AVI/PAV, and PAV/PAV).

Ordinal regression models (MASS package in R), adjusted for sex and age, were used to investigate the association between ARTIQ symptom severity and *TAS1R2, TAS1R3* SNPs, and *TAS2R38* haplotype. Specifically, as a first step, a basic model with only sex and age as covariates was applied (named “Model 0”). Then, a model including the haplotype or SNP was implemented (“Model 1”). The likelihood ratio test was used to assess the goodness of fit of the two models. All *p*-values were adjusted using the Benjamini–Hochberg method. An adjusted *p*-value of <0.05 was considered statistically significant.

All statistical analyses were performed using the R software version 4.1.2 (R Foundation for Statistical Computing, Vienna, Austria).

## 3. Results

### 3.1. COVID-19 Patient Clinical Evaluation

One hundred and ninety-six adult patients affected by COVID-19 were enrolled in this study. 

Complete demographic data and ARTIQ evaluation results are reported in Table 1.

The mean age of this cohort was 48.8 years and the majority of participants were women (137 out of 196 patients were female (69.9%)). 

Each patient underwent a careful assessment of COVID-19-related symptoms through the ARTIQ evaluation. In particular, according to the data collected, the severity of COVID-19 symptoms was evaluated as mild to moderate.

During the AP of infection, the most frequently reported symptoms were muscle pain (68.9%), joint pain (67.3%), and fever (65.8%). Other commonly reported symptoms were headache (59.7%), dry cough (53.6%), and blocked nose (53.6%), while hoarseness was observed in 49 out of 196 participants, and only 23 out of 196 described swollen glands.

### 3.2. TAS2R38, TAS1R2, and TAS1R3 Haplotype/Genotype Distribution

The *TAS2R38* haplotype distribution of this study cohort is reported in Table 2. In particular, AVI/AVI individuals represented 20.4% of the cohort, while PAV/AVI and PAV/PAV individuals represented 52.6% and 19.4% of the sample, respectively.

Regarding *TAS1Rs*, genetic analyses results for rs307355 of *TAS1R3* gene identified that the majority of the subjects (88.3%) were homozygous for the wild-type C allele (C/C genotype), 23 individuals were heterozygous carriers, while none of them were homozygous for the reference T allele (T/T genotype).

Concerning rs35874116 of the *TAS1R2* gene, the genetic analysis highlighted that 50.0% of patients were homozygous carriers of the wild-type T allele (T/T genotype), 39.8% were heterozygous C/T, and 20 participants were homozygous for the alternative C allele (C/C genotype) (Table 2).

### 3.3. Genetic Variation within TAS1R2, TAS1R3 and COVID-19 Symptoms

Complete results of the associations between the investigated *TAS1R2* and *TAS1R3* SNPs and the presence of COVID-19 symptoms, and their relative severity, are reported, respectively, in Tables S1 and S2.

Two statistically significant associations between rs307355 of the *TAS1R3* gene and COVID-19 symptom severity were detected (Table 3). In particular, both logistic and ordinal regression models revealed a positive association between rs307355 and two COVID-19-related symptoms: (i) chest pain and (ii) shortness of breath.

Specifically, in the AP of infection, patients carrying the C/C genotype showed an 8.11 times higher risk of manifesting chest pain (OR 8.11, 95% CI 2.26–51.99), and a 5.45 times higher risk of developing shortness of breath (OR 5.45, 95% CI 1.94–19.48) in comparison to heterozygous T/C carriers.

Moreover, regarding COVID-19 symptom severity, ordinal regression models revealed that homozygous C/C patients had an 8.30 times higher risk of manifesting severe chest pain (OR 8.30, 95% CI 2.32–53.13), and a 4.83 times higher risk of developing severe shortness of breath (OR 4.83, 95% CI 1.71–17.32) than heterozygous T/C carriers.

**Figure 1:**
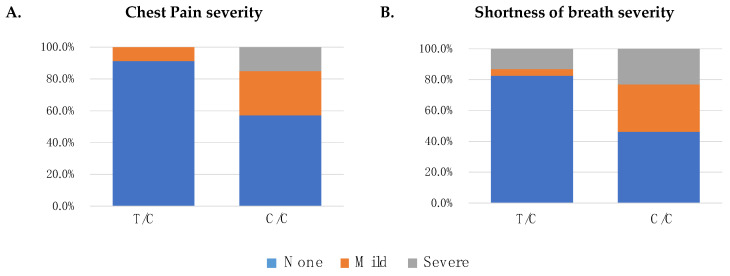
Chest pain and shortness of breath severity assessment and rs307355 (*TAS1R3* gene) genotype distribution. Graphical representation of chest pain (panel **A**) and shortness of breath (panel **B**) severity assessment according to rs307355 genotype distribution.

Figure 1 shows the distribution of the presence and severity of chest pain (A) and shortness of breath (B) symptoms. In particular, 91.3% (n = 21) of the heterozygous T/C patients showed no chest pain, 8.7% (n = 2) rated it as “Mild”, while no one reported “Severe” symptomatology. Conversely, chest pain was rated as “None” in 57.2% (n = 99), “Mild” in 27.7% (n = 48), and “Severe” in 15.0% (n = 26) of homozygous patients for the C allele.

Regarding shortness of breath, 82.6% (n = 19) of the heterozygous T/C carriers described the absence of this symptom, 4.3% (n = 1) rated it as “Mild”, and 13.0% (n = 3) rated it as “Severe”. Conversely, shortness of breath was rated as “None” in 46.2% (n = 80), “Mild” in 30.6% (n = 53), and “Severe” in 23.1% (n = 40) of the homozygous C/C patients.

### 3.4. Association between the TAS2R38 Haplotype and COVID-19 Symptoms

In order to investigate possible associations between the *TAS2R38* haplotype and COVID-19 symptoms and severity, logistic and ordinal regression models were used. 

None of the regression models performed provided statistically significant results, as reported in Tables S3 and S4. 

Overall, in this study, the results of statistical analyses highlighted an absence of association between *TAS2R38* haplotype and COVID-19 symptoms and severity.

## 4. Discussion

An intriguing parallelism between taste and immune system function is currently emerging. Specifically, as taste receptors play a key role in safeguarding against the ingestion of potentially detrimental substances, the immune system acts to “sense” and perceive the environment, protecting the human body from pathogens. Therefore, it is not unexpected that several mechanisms of human innate immunity are based on components of taste signal transduction [7]. In this line, several studies in the literature have reported that these two systems can interact and influence each other [37]; in fact, taste alterations have been linked with several inflammatory and autoimmune disorders (e.g., systemic lupus erythematosus and Sjögren’s syndrome) [38] and upper respiratory infections, with COVID-19 as a representative case.

The identification of extra-oral *TAS2Rs* and *TAS1Rs* enabled the characterisation of their additional role in innate immunity, being able to identify pathogens and trigger downstream responses within a few minutes [25]. Currently, little is known about *TAS1Rs’* role in immune response regulation, while *TAS2Rs’* contribution has been recently characterised. In particular, *TAS2R38* is the member of *TAS2Rs* family that is mainly involved in respiratory tract innate immune response. Specifically, TAS2R38 activation in response to AHLs regulates the release of AMPs and NO during infections [17]. Interestingly, it was demonstrated that NO inhibits the replication of SARS-CoV family viruses, impairing the binding between the spike protein and its receptor, angiotensin-converting enzyme 2, and reducing viral RNA synthesis in the early steps of replication [18].

In line with this evidence, a possible correlation between *TAS2R38* activity and COVID-19 immune response has been suggested, and a few studies have been carried out, with conflicting results [15]. Therefore, the relationship between *TAS2Rs*, *TAS1Rs* and SARS-CoV-2 infection is still an open question.

To fill this knowledge gap, this study took advantage of clinical and genetic data of a characterised cohort of 196 COVID-19 patients with mild-to-moderate symptoms. The availability of a detailed evaluation of COVID-19 symptoms, relative severity assessment, and *TAS2R38* and *TAS1R2/3* genetic analyses allowed us to (1) clarify the role of *TAS2R38* in determining COVID-19 symptom severity, (2) investigate, for the first time, the role of *TAS1Rs* in the immune response to viral infections.

Regarding the first aim, none of the regression models performed could generate statistically significant results between *TAS2R38* haplotype and the presence/severity of COVID-19 symptoms. Therefore, these results are in line with the only available study in the literature that genetically investigated the *TAS2R38* haplotype in COVID-19 patients, demonstrating an absence of association between the *TAS2R38* haplotype and COVID-19 symptoms/severity. 

Concerning the second aim, both logistic and ordinal regression models allowed the identification of two statistically significant positive associations between rs307355 of the *TAS1R3* gene and two COVID-19-related symptoms: (i) chest pain and (ii) shortness of breath. Specifically, in the AP of infection, patients carrying the C/C genotype showed an increased risk of manifesting severe forms of chest pain (OR 8.11, 95% CI 2.26–51.99) and shortness of breath (OR 4.83, 95% CI 1.71–17.32) in comparison with heterozygous T/C carriers.

As previously discussed, the knowledge regarding *TAS1Rs’* role in mediating viral innate immune response and COVID-19 symptom severity is far from complete; therefore, a replication of our findings in independent cohorts and functional studies could be instrumental in strengthening the reliability of these data. However, in view of these results, the *TAS1R3* gene could be a promising candidate to be investigated.

To date, it is widely recognised that TAS1R3 negatively regulates TAS2Rs activity depending on the airway surface liquid (ASL) glucose concentration [14]. In healthy conditions, there are low glucose levels in the ASL; these levels are sufficient to trigger TAS1R3 activation and inhibit TAS2Rs-mediated AMPs secretion. Conversely, during acute infections, rapid ASL glucose depletion by pathogens inhibits TAS1R3, thus allowing TAS2Rs to mediate an appropriate defence response [6,14]. Indeed, excessive NO production or AMPs release can have local harmful effects on the upper respiratory tract epithelium.

To support this tight regulation, some studies reported that, in patients affected by chronic rhinosinusitis (CRS), the ASL glucose concentration is three to four times higher than in healthy individuals, likely resulting from the disruption of tight junctions in human sinonasal cells caused by chronic infections. The increased levels of ASL glucose in CRS patients may be responsible for a continuous repression of TAS2Rs-mediated AMP secretion, thus impairing an effective immune response [11].

However, in this study, the two significant associations detected (i.e., chest pain and shortness of breath) are COVID-19 symptoms related to lower airway compartments.

To date, no studies have described *TAS1R3* gene function in regulating immune response or determining viral infection symptom severity in the human lower respiratory tract. However, considering the coupled activity of TAS1R3 with TAS2Rs, it cannot be excluded that TAS1R3 could be involved in regulating defence against pathogens in other body areas beyond the upper airways.

Indeed, in mice models, it has been reported that TAS2Rs are expressed in tracheal chemosensory cells, in which they regulate breath-holding responses in response to AHLs, and in bronchial smooth muscle cells, where they mediate bronchodilation [11].

Therefore, it can be speculated that alterations in TAS1Rs activity could hamper the function of TAS2Rs, thus leading to a worsening of chest pain and shortness-of-breath symptoms, as observed in this study cohort. Hence, further functional studies with in vitro/in vivo models will be fundamental to shed light on these mechanisms, thus offering novel insights into immune response regulation in the lower airways. Furthermore, since the study cohort comprised COVID-19 patients with mild-to-moderate symptoms, it would be useful to validate these findings in patients who are affected by more severe forms as well. 

Indeed, a complication of COVID-19 is acute respiratory distress syndrome (ARDS) [39], a disease characterised by increased pulmonary vascular permeability and reduced microvascular barrier integrity [40].

Interestingly, it has been demonstrated that TAS1R3 regulates the lung microvascular endothelial barrier function during ARDS manifestation [40]. In particular, in the pulmonary vasculature, in the presence of barrier-disruptive agents, the expression of *TAS1R3* is reduced, while exposure of lung microvascular endothelial cells to sweet compounds reduces the severity of endothelial barrier dysfunction.

In light of this, *TAS1R3* could be considered as a novel therapeutic target to treat ARDS, thus suggesting a wider role for this candidate in pulmonary-related symptoms in the context of SARS-CoV-2 infection [41].

## 5. Conclusions

In conclusion, this study, relying on a careful evaluation of COVID-19 associated symptoms and genetic analyses, allowed us to (1) exclude the role of *TAS2R38* in determining COVID-19 symptom severity, (2) gain novel insights on the impact of the *TAS1R3* gene on COVID-19 symptom severity and immune response.

Hence, a deeper understanding of the antiviral immune pathways associated with extra-oral taste receptors could pave the way for novel therapeutic strategies for COVID-19, independent of *TAS2R38* activity, aimed at (1) improving the TAS2R-mediated response, and (2) reducing TAS1Rs inhibition of TAS2Rs activity.

## Figures and Tables

**Table 1:** Demographic data and ARTIQ symptoms evaluation of COVID-19 patients. The table reports the main demographic and ARTIQ assessment data described as mean and standard deviation (Mean ± sd) for continuous variables, and as frequency and percentage (N (%)) for categorical variables. The presence of symptoms was registered as a dichotomous variable (1: “yes”/0: “no”). ARTIQ evaluation: presence of COVID-19-related symptoms, evaluated with the Acute Respiratory Tract Infection Questionnaire (ARTIQ).

Demographic and Clinical Data (n = 196)	Mean ± sd or N (%)
Age	48.8 ± 13.8
Gender	
Female	137 (69.9%)
Male	59 (30.1%)
Diabetes (n = 194)	6 (3.1%)
Cardiovascular disease (n = 194)	11 (5.7%)
Active cancer (n = 194)	2 (1.0%)
Chronic respiratory (n = 194)	6 (3.1%)
Liver disease (n = 194)	1 (0.5%)
**ARTIQ evaluation**	
Dry cough	105 (53.6%)
Coughing up mucus	50 (25.5%)
Hearing loss	56 (35.4%)
Blocked nose	105 (53.6%)
Rhinorrhoea	83 (52.9%)
Sneezing	83 (52.5%)
Lacrimation	61 (38.6%)
Hoarseness	49 (31%)
Fever	129 (65.8%)
Swelling	64 (40.8%)
Chills	81 (51.3%)
Headache	117 (59.7%)
Sore throat	83 (42.3%)
Muscle pain	135 (68.9%)
Joint pain	132 (67.3%)
Chest pain	76 (38.8%)
Sinonasal pain	54 (27.6%)
Swollen glands	23 (14.6%)
Loss of appetite	86 (43.9%)
Breathing problems	74 (37.8%)
Shortness of breath	97 (49.5%)

**Table 2:** *TAS2R38*, *TAS1R3*, *TAS1R2* haplotype/genotype distribution in the COVID-19 study cohort. The table reports TAS2R38 haplotype distribution and *TAS1R3* (rs307355) and *TAS1R2* (rs35874116) genotype distribution described as frequency and percentage (N (%)).

Genetic Analyses		N (%)
*TAS2R38* haplotype distribution		
	PAV/PAV	38 (19.4%)
	PAV/AVI	103 (52.6%)
	AVI/AVI	40 (20.4%)
Others		15 (7.7%)
	AAI/PAV	2 (1.02%)
	AAV/PAV	9 (4.6%)
	AVI/AAV	4 (2.04%)
*TAS1R3* (rs307355) genotype frequencies		
	T/T	0 (0.00%)
	T/C	23 (11.7%)
	C/C	173 (88.3%)
*TAS1R2* (rs35874116) genotype frequencies		
	T/T	98 (50.0)
	T/C	78 (39.8)
	C/C	20 (10.2)

**Table 3:** Statistically significant associations between rs307355 (*TAS1R3* gene) and COVID-19 symptoms and relative severity. This table reports the statistically significant results of logistic and ordinal regression models used to evaluate the association between presence of COVID-19 symptoms and relative severity and the analysed *TAS1R2/3* SNPs. The presence of COVID-19-related symptoms was registered as a dichotomous variable (1: “yes”/0: “no”), while COVID-19-related symptoms severity was ranked on a 0–2-point scale, as none (0), mild (1), severe (2). OR: Odds Ratio. CI: Confidence Interval 95%. All models are adjusted for sex and age.

COVID-19 Symptoms	Regression Models	rs307355 [C/C Genotype]		
		OR	CI 95%	*p*-Value
Chest Pain	Logistic	8.11	2.26–51.99	0.001
	Ordinal	8.30	2.32–53.13	0.0004
Shortness of breath	Logistic	5.45	1.94–19.48	0.001
	Ordinal	4.83	1.71–17.32	0.0021

## Data Availability

The data underlying this article will be shared on reasonable request to the corresponding author.

## Supplementary Materials

The following supporting information can be downloaded at: https://www.mdpi.com/article/10.3390/life14020219/s1, Table S1. Associations between rs35874116 (*TAS1R2* gene), rs307355 (*TAS1R3* gene) and COVID-19 symptoms, Table S2. Associations between rs35874116 (*TAS1R2* gene), rs307355 (*TAS1R3* gene) and COVID-19 symptom severity, Table S3. Associations between *TAS2R38* haplotype and COVID-19 symptoms, Table S4. Associations between *TAS2R38* haplotype and COVID-19 symptom severity, Table S5. Abbreviations and acronyms.
