# Supplementary material for: The Bittersweet Symphony of COVID-19: Associations between TAS1Rs and TAS2R38 Genetic Variations and COVID-19 Symptoms
Source: Life (Basel). 2024 Feb 3;14(2):219. doi: 10.3390/life14020219 (PMC10890446; doi:10.3390/life14020219)
Supplement: Supplementary file 1 [file life-14-00219-s001.zip › Table S1_sweet receptors_logistic models.pdf]

Table S1. **Associations between rs35874116 (*TAS1R2* gene), rs307355 (*TAS1R3* gene) and COVID-19 symptoms.** Presence of COVID-19-related symptoms was registered as a dichotomous variable (1:"yes"/0:"no"). P-value columns report not adjusted *p*-values. Statistically significant models (Benjamini-Hochberg *adjusted* *p*-value<0.05) are in bold. OR: Odds Ratio. 95% CI: 95% Confidence Interval.

**COVID-19 symptoms**	**rs35874116 (*TAS1R2*)**			**rs307355 (*TAS1R3*)**		
	**OR**	**95% CI**	***p*-value**	**OR**	**95% CI**	***p*-value**
Smell taste	0.76	(0.45 - 1.33)	0.3359	1.20	(0.37 - 3.33)	0.749
Dry cough	1.06	(0.69 - 1.62)	0.7977	1.03	(0.42 - 2.48)	0.956
Coughing up mucus	0.87	(0.52 - 1.42)	0.5718	2.32	(0.74 - 10.25)	0.157
Hearing loss	0.80	(0.48 - 1.31)	0.3864	1.48	(0.52 - 4.85)	0.472
Blocked nose	0.88	(0.57 - 1.34)	0.5423	1.84	(0.76 - 4.67)	0.178
Rhinorrhea	1.12	(0.70 - 1.81)	0.6349	1.37	(0.5 - 3.83)	0.540
Sneezing	1.03	(0.65 - 1.66)	0.8901	1.44	(0.53 - 4)	0.474
Lacrimation	1.08	(0.66 - 1.73)	0.7616	2.47	(0.83 - 9.12)	0.107
Raucousness	0.99	(0.59 - 1.64)	0.9802	1.61	(0.54 - 5.94)	0.414
Fever	1.13	(0.72 - 1.79)	0.6083	0.67	(0.23 - 1.73)	0.425
Swelling	1.46	(0.91 - 2.37)	0.1202	1.88	(0.66 - 6.16)	0.242
Chills	1.17	(0.73 - 1.88)	0.5175	2.97	(1.05 - 9.74)	0.040
Headache	1.06	(0.68 - 1.66)	0.8037	3.00	(1.21 - 7.97)	0.018
Sore throat	1.30	(0.84 - 2.02)	0.2395	1.63	(0.65 - 4.49)	0.303
Muscle pain	0.95	(0.61 - 1.51)	0.8373	1.46	(0.57 - 3.56)	0.419
Joint pain	0.90	(0.57 - 1.41)	0.6395	2.03	(0.83 - 4.96)	0.120
**Chest pain**	1.46	(0.94 - 2.27)	0.0889	**8.11**	**(2.26 - 51.99)**	**0.001**
Sinonasal pain	0.80	(0.48 - 1.29)	0.3623	1.80	(0.63 - 6.48)	0.288
Neck tumefaction	1.18	(0.61 - 2.23)	0.6121	3.22	(0.60 - 59.68)	0.198
Loss of appetite	0.96	(0.62 - 1.48)	0.8611	1.42	(0.58 - 3.73)	0.450
Breathing problems	1.03	(0.66 - 1.6)	0.9006	3.12	(1.1 - 11.21)	0.031
**Shortness of breath**	1.19	(0.78 - 1.83)	0.4241	**5.45**	**(1.94 - 19.48)**	**0.001**
