# Supplementary material for: The Bittersweet Symphony of COVID-19: Associations between TAS1Rs and TAS2R38 Genetic Variations and COVID-19 Symptoms
Source: Life (Basel). 2024 Feb 3;14(2):219. doi: 10.3390/life14020219 (PMC10890446; doi:10.3390/life14020219)
Supplement: Supplementary file 1 [file life-14-00219-s001.zip › Table S2_sweet receptors_ordinal models.pdf]

Table S2. **Associations between rs35874116 (*TAS1R2* gene), rs307355 (*TAS1R3* gene) and COVID-19 symptoms severity.** COVID-19-related symptoms severity was ranked on a 0–2-point scale. P-value columns report not adjusted *p*-values. Statistically significant models (Benjamini-Hochberg *adjusted p*-value<0.05) are in bold. OR: Odds Ratio. 95% CI: 95% Confidence Interval.

**COVID-19 symptoms severity**	**rs35874116 (*TAS1R2*)**		**rs307355 (*TAS1R3*)**	
	**OR (95% CI)**	***p*-value**	**OR (95%CI)**	***p*-value**
Smell taste	0.96 (0.61 - 1.52)	0.8485	0.95 (0.34 - 2.36)	0.9091
Dry cough	1.00 (0.68 - 1.47)	0.9966	1.15 (0.52 - 2.64)	0.7326
Coughing up mucus	0.83 (0.50 - 1.35)	0.4571	2.22 (0.71 - 9.78)	0.1847
Hearing loss	0.77 (0.46 - 1.27)	0.3096	1.53 (0.55 - 4.94)	0.4271
Blocked nose	0.89 (0.59 - 1.33)	0.5657	1.92 (0.83 - 4.72)	0.1288
Rhinorrhea	1.16 (0.74 - 1.82)	0.5208	1.42 (0.56 - 3.82)	0.4664
Sneezing	1.09 (0.69 - 1.73)	0.7023	1.11 (0.41 - 3.11)	0.8351
Lacrimation	1.12 (0.69 - 1.78)	0.6497	2.40 (0.81 - 8.85)	0.1168
Raucousness	0.94 (0.57 - 1.54)	0.8169	1.55 (0.52 - 5.74)	0.4464
Fever	1.04 (0.70 - 1.53)	0.8483	0.80 (0.36 - 1.76)	0.5813
Swelling	1.38 (0.87 - 2.19)	0.1723	1.74 (0.63 - 5.65)	0.2984
Chills	1.21 (0.77 - 1.90)	0.4146	2.47 (0.87 - 8.09)	0.0902
Headache	0.90 (0.61 - 1.34)	0.6074	3.06 (1.28 - 7.95)	0.0114
Sore throat	1.39 (0.91 - 2.13)	0.1228	1.20 (0.47 - 3.34)	0.7035
Muscle pain	0.94 (0.63 - 1.40)	0.7548	1.42 (0.63 - 3.23)	0.3913
Joint pain	0.88 (0.58 - 1.31)	0.5176	2.26 (1.01 - 5.20)	0.0459
**Chest pain**	1.38 (0.91 - 2.09)	0.1256	**8.30 (2.32 - 53.13)**	**0.0004**
Sinonasal pain	0.80 (0.49 - 1.30)	0.3772	1.86 (0.66 - 6.65)	0.257
Neck tumefaction	1.21 (0.62 - 2.28)	0.5583	3.04 (0.57 - 56.41)	0.2236
Loss of appetite	0.97 (0.63 - 1.47)	0.8763	1.30 (0.54 - 3.37)	0.5692
Problems breathing	1.01 (0.66 - 1.54)	0.9624	3.02 (1.07 - 10.81)	0.0353
**Shortness of breath**	1.13 (0.76 - 1.69)	0.5437	**4.83 (1.71 - 17.32)**	**0.0021**
