# Supplementary material for: The Bittersweet Symphony of COVID-19: Associations between TAS1Rs and TAS2R38 Genetic Variations and COVID-19 Symptoms
Source: Life (Basel). 2024 Feb 3;14(2):219. doi: 10.3390/life14020219 (PMC10890446; doi:10.3390/life14020219)
Supplement: Supplementary file 1 [file life-14-00219-s001.zip › Table S3_TAS2R38_logistic models.pdf]

Table S3. Associations between *TAS2R38* haplotype and COVID-19 symptoms. Presence of COVID-19-related symptoms was registered as a dichotomous variable (1:"yes"/0: no"). All models are adjusted for sex and age. OR: Odds Ratio. 95% CI: 95% Confidence Interval.

COVID-19 symptoms	OR (95% CI) - AVI/PAV	OR (95% CI) - PAV/PAV	*p*-value
Smell taste	0.78 (0.27 - 2.06)	1.17 (0.32 - 4.32)	0.70
Dry cough	0.77 (0.36 - 1.61)	1.15 (0.46 - 2.90)	0.53
Coughing up mucus	1.19 (0.51 - 2.99)	1.96 (0.71 - 5.62)	0.38
Hearing loss	0.97 (0.40 - 2.38)	1.39 (0.49 - 4.02)	0.70
Blocked nose	0.83 (0.39 - 1.75)	0.80 (0.32 – 2.00)	0.87
Rhinorrhea	1.16 (0.50 - 2.71)	1.31 (0.47 - 3.71)	0.87
Sneezing	1.15 (0.50 - 2.64)	1.25 (0.45 - 3.48)	0.91
Lacrimation	0.91 (0.39 - 2.21)	1.57 (0.56 - 4.47)	0.45
Raucousness	1.23 (0.50 - 3.21)	1.52 (0.51 - 4.61)	0.76
Fever	0.94 (0.43 - 2.03)	1.33 (0.50 - 3.64)	0.72
Swelling	0.88 (0.38 - 2.09)	0.84 (0.29 - 2.36)	0.94
Chills	0.94 (0.40 - 2.17)	0.97 (0.35 - 2.71)	0.99
Headache	1.56 (0.72 - 3.39)	1.44 (0.56 - 3.74)	0.52
Sore throat	0.83 (0.39 - 1.77)	0.98 (0.39 - 2.46)	0.86
Muscle pain	1.43 (0.65 - 3.10)	1.51 (0.58 - 4.03)	0.62
Joint pain	1.50 (0.69 - 3.21)	1.44 (0.56 - 3.76)	0.58
Chest pain	0.96 (0.45 - 2.09)	1.11 (0.44 - 2.83)	0.93
Sinonasal pain	1.51 (0.66 - 3.76)	1.90 (0.69 - 5.42)	0.45
Neck tumefaction	1.20 (0.37 - 4.70)	2.36 (0.61 - 10.24)	0.38
Loss of appetite	0.46 (0.21 - 0.98)	0.55 (0.22 - 1.38)	0.13
Problems breathing	0.92 (0.44 - 1.97)	0.55 (0.20 - 1.43)	0.39
Shortness of breath	0.88 (0.41 - 1.83)	0.51 (0.20 - 1.28)	0.30
