# Supplementary material for: The Bittersweet Symphony of COVID-19: Associations between TAS1Rs and TAS2R38 Genetic Variations and COVID-19 Symptoms
Source: Life (Basel). 2024 Feb 3;14(2):219. doi: 10.3390/life14020219 (PMC10890446; doi:10.3390/life14020219)
Supplement: Supplementary file 1 [file life-14-00219-s001.zip › Table S4_TAS2R38_ordinal models.pdf]

Table S4. Associations between *TAS2R38* haplotype and COVID-19 symptoms severity. COVID-19-related symptoms severity was ranked on a 0–2-point scale, as none (0), mild (1), severe (2). All models are adjusted for sex and age. OR: Odds Ratio. 95% CI: 95% Confidence Interval.

**ARTIQ symptoms severity**	**OR AVI/PAV (95% CI)**	**OR PAV/PAV (95% CI)**	***p*-value**
Smell taste	0.86 (0.37 - 1.89)	1.65 (0.58 - 4.97)	0.3312
Dry cough	0.94 (0.47 - 1.87)	1.11 (0.49 - 2.52)	0.8954
Coughing up mucus	1.16 (0.50 - 2.91)	1.70 (0.63 - 4.79)	0.5393
Hearing loss	0.97 (0.41 - 2.36)	1.14 (0.41 - 3.18)	0.9303
Blocked nose	0.70 (0.35 - 1.41)	0.59 (0.25 - 1.38)	0.4571
Rhinorrhea	1.04 (0.47 - 2.30)	1.28 (0.49 - 3.41)	0.8436
Sneezing	1.20 (0.55 - 2.67)	1.14 (0.44 - 2.94)	0.9029
Lacrimation	0.96 (0.41 - 2.30)	1.40 (0.52 - 3.84)	0.6508
Raucousness	1.15 (0.47 - 2.99)	1.32 (0.45 - 3.97)	0.8798
Fever	0.63 (0.31 - 1.27)	0.98 (0.41 - 2.32)	0.2694
Swelling	0.92 (0.41 - 2.12)	0.76 (0.28 - 2.06)	0.8561
Chills	0.83 (0.37 - 1.84)	0.68 (0.26 - 1.77)	0.7304
Headache	1.34 (0.67 - 2.70)	1.49 (0.63 - 3.52)	0.6203
Sore throat	1.06 (0.53 - 2.16)	1.19 (0.49 - 2.82)	0.9306
Muscle pain	1.12 (0.56 - 2.25)	1.08 (0.47 - 2.52)	0.9509
Joint pain	1.25 (0.62 - 2.49)	1.11 (0.48 - 2.56)	0.8175
Chest pain	1.00 (0.47 - 2.14)	0.98 (0.40 - 2.41)	0.9983
Sinonasal pain	1.40 (0.61 - 3.48)	1.62 (0.60 - 4.55)	0.6208
Neck tumefaction	1.26 (0.38 - 4.89)	2.39 (0.62 - 10.29)	0.3909
Loss of appetite	0.49 (0.24 - 0.99)	0.46 (0.19 - 1.07)	0.1027
Breathing problems	0.94 (0.46 - 1.96)	0.50 (0.19 - 1.26)	0.2367
Shortness of breath	0.93 (0.47 - 1.86)	0.50 (0.20 - 1.17)	0.1869
