# Supplementary material for: The Bittersweet Symphony of COVID-19: Associations between TAS1Rs and TAS2R38 Genetic Variations and COVID-19 Symptoms
Source: Life (Basel). 2024 Feb 3;14(2):219. doi: 10.3390/life14020219 (PMC10890446; doi:10.3390/life14020219)
Supplement: Supplementary file 1 [file life-14-00219-s001.zip › Table S5_Abbreviations and acronyms.pdf]

Table S5. **Abbreviations and acronyms.** An alphabetic list of all the abbreviations and acronyms reported in the manuscript is listed in the left column; their full meaning is reported in the right column.

Abbreviations and acronyms	Full meaning
Acute phase	AP
Acute Respiratory Distress Syndrome	ARDS
Acute Respiratory Tract Infection Questionnaire	ARTIQ
Acyl-Homoserine Lactones	AHLs
Airway surface liquid	ASL
Alanine-Valine-Isoleucine	AVI
Antimicrobial peptides	AMPs
Bitter taste receptors	TAS2Rs
Chronic rhinosinusitis	CRS
COVID-19	Coronavirus disease 2019
G-protein-coupled receptors	GPCRs
Nitric oxide	NO
Phenylthiocarbamide	PTC
Polymerase chain reaction	PCR
Proline-Alanine-Valine	PAV
Propylthiouracil	PROP
SARS-CoV-2	Severe Acute Respiratory Syndrome Coronavirus 2
Single Nucleotide Polymorphisms	SNPs
Solitary chemosensory cells	SCCs
Sweet taste receptors	TAS1Rs
Taste-sensing type 2 receptor	TAS2R38
Whole-Genome sequencing	WGS
